# Intelligence IS Cognitive Flexibility: Why Multilevel Models of Within-Individual Processes Are Needed to Realise This

**DOI:** 10.3390/jintelligence10030049

**Published:** 2022-08-01

**Authors:** Damian P. Birney, Jens F. Beckmann

**Affiliations:** 1 School of Psychology, University of Sydney, Sydney 2006, Australia; 2 School of Education, Durham University, Durham DH1 1TA, UK; j.beckmann@durham.ac.uk

**Keywords:** cognitive flexibility, ergodic assumption, formative models, multilevel models, complex problem-solving

## Abstract

Despite substantial evidence for the link between an individual’s intelligence and successful life outcomes, questions about what defines intelligence have remained the focus of heated dispute. The most common approach to understanding intelligence has been to investigate what performance on tests of intellect is and is not associated with. This psychometric approach, based on correlations and factor analysis is deficient. In this review, we aim to substantiate why classic psychometrics which focus on between-person accounts will necessarily provide a limited account of intelligence until theoretical considerations of within-person accounts are incorporated. First, we consider the impact of entrenched psychometric presumptions that support the status quo and impede alternative views. Second, we review the importance of process-theories, which are critical for any serious attempt to build a within-person account of intelligence. Third, features of dynamic tasks are reviewed, and we outline how static tasks can be modified to target within-person processes. Finally, we explain how multilevel models are conceptually and psychometrically well-suited to building and testing within-individual notions of intelligence, which at its core, we argue is cognitive flexibility. We conclude by describing an application of these ideas in the context of microworlds as a case study.

## 1. Introduction

One of the *least* disputed claims in psychology is the link between an individual’s intelligence and successful life outcomes, particularly in academia and work (Gottfredson 1997, 2018; Mackintosh 2011; Sternberg et al. 2000). Paradoxically, some of the *most* disputed claims in psychology concern how to define and operationalise intelligence (Gottfredson 2018; Horn and Noll 1994). The solution to the definition-operationalisation problem has less to do with filling some sparsity of theorising, there is much to draw from (Sternberg 2020). Instead, we are hamstrung by psychometric methods that are at once too flexible, too constrained, and too disconnected from substantive theory. We advocate for approaches to intelligence that are directed at within-individual processes, rather than at between-individual comparisons because they are fundamentally closer to the conceptual notion of adaptivity. Adaptivity in complex and novel situations requires rapid and flexible encoding, representation, and manipulation of relations between aspects of the physical and mental world (Beckmann 2014). Our aim in this review is first to explicate a notion of intelligence in which the conceptualisation *and* operationalisation are jointly integrated, aligned, and directly related to how one successfully adapts to changing demands of the environment or task from one situation to the next. Second, we aim to demonstrate why a multilevel, analytic framework is critical to achieve this. To distinguish our notion from the status quo, particularly ‘g’ and Fluid Intelligence (*Gf*), we use the term “intelligence as cognitive flexibility”. We do so more as a placeholder because if it had not already lost most of its meaning (Gottfredson 2018), the term *intelligence* would better serve our intentions.

The focus on managing changing demand is consistent with common definitions of fluid intelligence, defined as entailing “deliberate but flexible control of attention to solve novel ‘on the spot’ problems that cannot be performed by relying exclusively on previously learned habits, schemas, and scripts” (Schneider and McGrew 2012, p. 111). Yet, whether one accepts this definition or another, in practice it is primarily between-individual accounts which dominate the operationalisation of virtually all variants of intelligence, *Gf* included. As we will argue, this first serves to relegate the identification of flexibility to unnecessarily indirect inference from tests that do not require adaptation whatsoever, and second, as demonstrated by others, it relies on a somewhat dubious extrapolation of the *ergodic assumption*, that causal inferences from between-individual models map directly on to within-individual mechanisms (Borsboom et al. 2003; Molenaar 2004, 2013).

The mechanisms of intelligence most theories draw on relates to those extensively studied by cognitive psychologists, such as memory, attention, switching, inhibitory control, and relational binding, as well as higher-order concepts such as working memory and reasoning. De Boeck et al. (2020) argue that while there was early promise in the decomposition of reasoning tasks into such component processes to investigate process correlates of intelligence (for instance, Sternberg 1977a, 1977b), these innovations were ultimately not pursued, in part because of the emerging domination of factor analysis in theory development. That is, while these cognitive psychology constructs tend to have articulated process accounts, they were not the panacea to the conceptualisation-operationalisation misalignment of intelligence hoped for. Translating these process-focused constructs into assessments, sometimes referred to as elementary cognitive tasks (ECTs), has psychometric challenges which the traditional latent variable (psychometric) approach to intellectual abilities cannot resolve alone (Goecke et al. 2021).

### Overview

In this review, we aim to substantiate why the classic psychometric approach will always necessarily provide a limited account of intelligence and what might be done to redress this. The paper is structured in four parts. In Part 1 we consider the implications of three common but theoretically dubious practices that have become entrenched and serve to reinforce the status quo while impeding alternative views and potential progress. In Part 2 we review the importance of process-theories, which are critical for any serious attempt to build a within-person account of intelligence. In Part 3 we explicate the distinction between typical *static* tasks and *dynamic* tasks, which are by design focused on within-individual processes, and outline how the former can be modified to approximate the latter. Finally, in Part 4 we explain how multilevel, mixed effects analytic approaches both are conceptually and psychometrically well-suited to building and testing within-individual notions of intelligence—to narrowing the theory-operationalisation gap. We conclude by describing an application of these ideas as a case study.

We reflect on these four aspects because they are relevant to any proposition that aims to explicate a more authentic and dynamic definition of intelligence. There is a subtle but important difference between a proposition that we should take dynamic processes seriously, and a claim that traditional psychometrics are not well suited to achieve this. We necessarily address these psychometric issues in Part 1 because they are, or at least should be, the pillars of operationalisation and measurement (Birney et al. 2022; Michell 1990).

## 2. Part 1: Building a Case for Intelligence as Cognitive Flexibility

### 2.1. Entrenched Assumptions

Across the course of the history of intelligence theorising, a number of presumptions have worked their way into the collective consciousness and are now considered “knowns” (Neisser et al. 1996). Many of these, we believe, have become largely dogmatic, unquestionable “facts”. We consider three; (a) the supposition of stability, (b) the belief that factor analysis of correlations alone can reveal *true* latent processes and attributes within the individual, and (c) the view that observed variables (i.e., test scores) must be manifestations of these latent processes, rather than seriously considering that tests scores are formative causes of latent variables. That these are typically assumptions necessary to simplify psychometric modelling, rather than being core, testable theoretical tenets, has been known for some time. A small but increasingly vocal collective are questioning not only the validity of these “knowns”, but also critically reflecting on the limitations of their utility in providing a greater understanding of intelligence (e.g., Bollen and Diamantopoulos 2017; Borsboom 2015; Conway et al. 2021; De Boeck et al. 2020; Kovacs and Conway 2016; Molenaar 2013; van der Maas et al. 2017).

#### 2.1.1. Supposition of Stability

Whereas personality assessments tend to focus on typical levels, intelligence tests aim at assessing maximal performance levels (Neisser et al. 1996). From this, Goff and Ackerman (1992, p. 538) suggested that the use of intelligence tests actually implies “the existence of a stable or permanent capability”. We are not arguing against the goal of assessing maximal performance, because it largely reflects what researchers and educators intentionally set out to assess going at least as far back as Binet (1905)—a correlate of a nascent aptitude or cognitive potential. However, the assumption of inherent stability as a psychometric criterion, realised by concepts like test–retest reliability, is ostensibly antithetical to the notion of within-individual variability, including learning and development, and over time this has led to a set of psychometric practices well-suited to stable attributes but not systematically varying ones. In other words, if the starting assumption for mapping the assessment of a given set of intellectual attributes is that there is no or minimal within-individual variability, then stability-focused assessment and validation methods will evolve accordingly. As a result, “successful” measurement, so defined, not only risks becoming dissociated from the conceptual understanding of cognitive capabilities, our conceptual understanding may be skewed to fit our measurement assumptions.

These types of limitations of traditional psychometrics have long been recognized as overly restrictive in areas where assessment of dynamic processes is of interest, for instance, Dynamic Testing (Grigorenko and Sternberg 1998; Guthke and Beckmann 2000), complex-problem solving (Beckmann et al. 2017; Dörner and Funke 2017), and more recently cognitive flexibility (Beckmann 2014). The point here is that the extant psychometric principles of best-test design are often challenged by constructs that are by definition dynamic, fluid, and complexly determined by transient or volatile contextual and intra-personal factors. This is what needs to be redressed.

#### 2.1.2. The Ergodic Assumption: History Tells Us Correlations Are Not Enough; Logic Tells Us They Never Were

The individual-differences approach to the investigation of psychological attributes generally, and intellectual abilities specifically, has long been known to be incomplete without a consideration of process-oriented accounts (Cronbach 1957; Deary 2001; van der Maas et al. 2017). Lohman and Ippel (1993, p. 41) citing Cronbach (1957), McNemar (1964), Spearman (1927) and others, concluded that a major reason why the individual differences approach to the study of intelligence “… was unable to achieve one of its central goals: the identification of mental processes that underlie intelligent functioning”, was because “… a research program dominated by factor analysis of test intercorrelations was incapable of producing an explanatory theory of human intelligence”.

In his presidential address to the annual meeting of the Psychometric Society, Guttman (1971) contrasted the purpose of observation in the psychometric testing tradition, which was (and generally still is) to compare individuals, with his proposed, amended purpose which was to assess the *structure of relationships among observations*. In effect, Guttman was arguing that if one wishes to better understand the processes of intelligence, one needs to take a distinctively within-individual perspective. Lohman and Ippel (1993, p. 42) went further and suggested that the general idea of test theory as applied statistics (i.e., psychometrics) has not only hampered the development of *structural theories* for the measurement of processes, but actually precluded it (see also, Deary 2001; Molenaar 2004). Borsboom et al. (2003) later made the compelling argument “that between-subjects models do not imply, test, or support causal accounts that are valid at the individual level.” (p. 214). Additionally, that therefore, within-individual level processing must be explicitly incorporated in measurement models in order to substantively link between-subject models of intellect with what is happening at the level of the individual (Borsboom et al. 2004). As we will elaborate on in a later section (Part 4), like others (e.g., De Boeck et al. 2020), we see promise in multilevel (mixed-effects) models (MLM) for linking theory and measurement.

The claim that the structure observed at a between-individual level exists at the level of an individual is referred to as the *Ergodic Assumption* (Molenaar 2004, 2013). As explicated formally by Molenaar (2004, 2013), when there is substantial heterogeneity across individuals, or in other words, when stationarity of means and covariances does not exist across time/occasions, as is true for biological systems, including that of humans, the likelihood of the ergodic assumption being true is vanishingly low. The implication of this for the current discussion (and the field in general) is that the majority of between-individual conceptualisations of intelligence, such as that represented by the Cattell-Horn-Carroll (CHC) hierarchical taxonomy (Carroll 1993; Schneider and McGrew 2012) of human abilities, probably do not hold for most individuals. It is conceivable to say, Damian’s inductive, quantitative, and verbal attributes (narrow CHC factors) covary differently relative to Jens’; that is, their CHC “factor structures” are different. When we assess between-person CHC factors, such as inductive reasoning, quantitative reasoning, and verbal comprehension, we are making the unstated supposition that each of these attributes exists uniquely within the person we are assessing. We are certainly doing so when we plot the person’s profile of derived scores as indices of CHC factors, and then interpret their strengths and weaknesses. This is precisely the ergodic assumption as it is realised in practice. In fact, Molenaar (2004, p. 215) concludes that for nonergodic processes “there is no scientifically respectable alternative but to study the structures of [within-individual variability] and [between-individual variability] for their own sake”. Of course, there are subdisciplines of researchers who have devoted considerable energies to each. Cronbach (1957) referred to them as *experimentalists* and *correlationalists* and argued that there will always remain questions that “Nature will never answer until our two disciplines ask [them] in a single voice” (p. 683).

#### 2.1.3. Ontological Status of Reflective vs. Causal- and Composite-Formative Concepts

The common factor-analytic/SEM model on which CHC is based is a *reflective* one, where individual differences in observed variables (and latent variables in hierarchical models) are considered *effect-indicators* of the latent attribute of interest^1^. That is, the variance in scores on the observed indicators represents *effects* that are *caused* by the latent variable. An alternative is to consider causal *formative* models, where observed variables (and latent variables) are *cause-indicators*. Here, variation in the resulting latent variable is *caused* by the indicators. Thus in formative models, the latent variable represents the indicators’ shared contribution in some collective way (Bollen and Diamantopoulos 2017; Kovacs and Conway 2016).

Formative models have typically not been broadly adopted by intelligence researchers (cf., Kovacs and Conway 2016), in spite of the fact they have been known since at least the 1960s (see Blalock, H.M, 1963, cited in Bollen and Diamantopoulos 2017). Bollen and Diamantopoulos (2017) suggest this is in part due to an historical entrenchment of thinking in terms of reflective models. This is not particularly surprising since theorisation is typically targeted at individual-centred processes that are intuitively reflective in nature, but such claims should be tested, not assumed. Bollen and Diamantopoulos review seven common criticism presented against the appropriateness of using formative indicators. They conclude each criticism is either invalid or represents issues shared by reflective indicators. Importantly for our purposes, the authors demarcate the difference between *causal*-formative and *composite*-formative indicators in terms of conceptual-unity, a distinction they argue is often ignored or misunderstood. When corrected, this leads to a straightforward discounting of the core criticisms and their basic tenets. Bollen and Diamantopoulos (2017) demonstrate that latent variables derived from models of causal-formative indicators which have what they refer to as *conceptual unity*, can be considered as measures^2^, analogous to reflective latent variables. Conceptual unity exists when each indicator matches “the idea embodied by the concept” (p. 584). How precisely this is achieved is not clear; it is an aspect of the theorising needing further explication. However, according to Bollen and Diamantopoulos, composite-formative indicators do not require conceptual unity, and therefore composite variables are not measures, they are not latent variables, and neither are the indicators causes of the composite variable. Composite variables may have utility as a summary of the multiple variables in a predictive sense but not an explanatory one.

**Figure 1:**
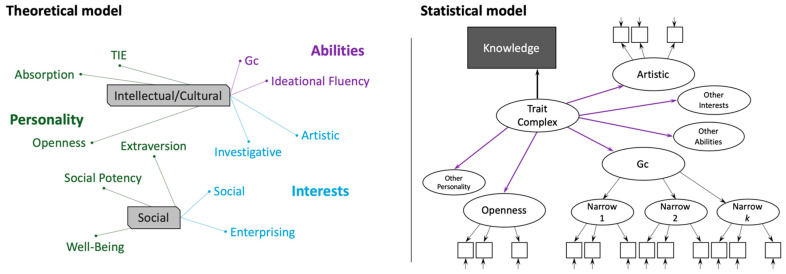
Schematic representation of the Intellectual/Cultural and Social trait-complexes proposed by Ackerman and Heggestad (1997). Left panel describes theoretical account; Right panel represents a reflective model of the intellectual/cultural trait-complex.

The demarcation between a composite vs. causal indicator is difficult to resolve. The identification of trait-complexes (Ackerman et al. 2013) present a potentially illustrative case in point. Ackerman and Heggestad (1997) proposed that there are four trait-complexes, two of which are represented in Figure 1 (left panel), that each encompass an overlapping set of different traits from the domains of personality, abilities, and interests (additional trait-complexes were subsequently included, see Ackerman et al. 2013). “Validity” of trait-complexes is purportedly evidenced by their differential prediction of domain-specific knowledge acquisition. For instance, the *intellectual/cultural* trait-complex was captured by *Gc* and ideational fluency abilities, artistic and investigative interests, and absorption, openness, and typical-intellectual engagement personality dimensions.

For instance, indicators of Openness (i.e., items) have conceptual unity necessary (but not sufficient) for measurement, because they are bound by the *definition* of the openness concept. However, although Ackerman et al. (2013) modelled trait-complexes as reflective latent traits as represented in Figure 1, it is reasonable to question whether they are formative (and therefore the purple arrows in Figure 1 should point to the trait-complex, rather than from it). If they are formative, then the next question is whether the indicators (i.e., personality, interests, and ability factors) together have sufficient conceptual unity necessary for the resulting trait-complexes to serve as latent variables (i.e., are causal-formative) or not (i.e., are composite-formative).

According to Bollen and Diamantopoulos (2017), while there are tests to determine whether a concept is likely reflective or formative, whether one treats a concept (such as a trait-complex) as causal- or composite-formative is an *ex ante* decision the researcher makes via an empirically substantiated theoretical claim^3^.

Our previous attempts at conceptualising cognitive flexibility as a meta-competency (Yu et al. 2019) has similar formative features. In this work, we surmised that there is a case for considering *cognitive flexibility as a meta-competency* to unify cognitive, conative (e.g., meta-cognitive) and situational dependencies, rather than thinking of cognitive flexibility simply as a facet of a broader flexibility attribute, as it is frequently conceived. Like the argument for trait-complexes, flexibility as a meta-competency is framed as a formative concept, but one that is probably composite in nature. The reason for classifying it as such, is that the theoretical boundaries for the meta-competency are still to be fully mapped and measurement properties still need to be better understood. Currently as it stands, while its indicators are internally coherent and (historically) considered reflective, as a set they lack sufficient conceptual unity.

**Figure 2:**
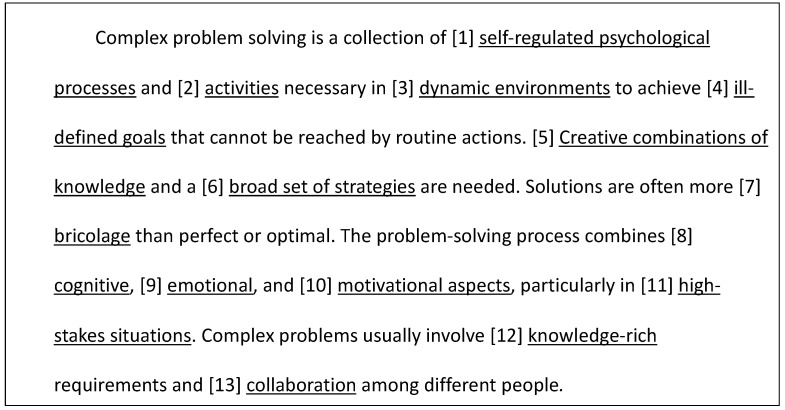
Excerpt from Dörner and Funke (2017, p. 6) showing distinct components (our enumeration and underlining) likely to define a composite-formative variable in the Bollen and Diamantopoulos (2017) framework.

The notion of Complex Problem Solving (Dörner and Funke 2017) also has many features of a composite-formative model. This is evident when one considers how it is conceptually defined, as demonstrated in the excerpt from Dörner and Funke (2017, p. 6) in Figure 2. We highlight 13 distinct components that relate to the theory of complex problem solving. Whether these components have sufficient conceptual unity to be anything other than composite-formative is not a statistical question, but rather intrinsically a theoretical *and* empirical one. That is, the ontological status cannot be assumed.

Thinking more broadly, one might further postulate that other “intelligences”, like practical intelligence or cultural intelligence, or even operational intelligence, coined by Dörner (1986, p. 290) in relation to complex problem-solving competencies, and defined as “the factors that determine the cognitive processes commonly labelled as flexibility, foresight, circumspection, systematic planning…”, are similarly defined conceptually with formative characteristics. This is not to disparage these or our own theories and models as being of lesser worth, it is simply being true to our understanding of the nature of the concept under investigation^4^. In summing up their commentary, Bollen and Diamantopoulos (2017) conclude that it does not matter too much whether the ontological basis of our theories are reflective or formative, the important scientific point is that researchers carefully “define their concept, choose corresponding indicators, and consider whether the *indicators depend on or influence* the latent variable” (p. 594, our emphasis). In our view ontological considerations are critical. This is because the proliferation of new latent variables unthinkingly assumed to be reflective, has obscured rather than illuminated our understanding of underlying processes.

To conclude this section, we make note of Process Overlap Theory (POT), a recent causal-formative account of intelligence (Kovacs and Conway 2016). According to Conway et al. (2021, p. 1) much of the motivation for POT is a growing dissatisfaction with the impediment to theory building caused by the disconnect between psychometrics and psychological theories, and problematic inferences related to the status of latent variables. Their argument is that the typical latent variable account, based in reflective SEM models where the latent variable is assumed to *causally determine* (i.e., is manifested in) individual differences in observed test scores, overlooks the real possibility that the emergence of a latent variable from such statistical approaches is an epiphenomenon of the fact that different tasks share different common processes, as represented statistically by causal-formative models. This is consistent with the work of van der Maas et al. (2006) who demonstrated that reciprocal mutualism between processes sufficiently explains positive manifold without the need to introduce a reflective latent attribute, such as ‘g’. Importantly however, Fried (2020) has demonstrated that network models are not necessarily differentiable from reflective models in terms of explained variance. Thus, simply moving to a formative account (or even a network one) is not sufficient. The burden now rests with the researcher to explicate the specific processes entailed.

### 2.2. Summary of Part 1: Why Intelligence Theorising Has Survived However, Failed to Thrive

In Part 1 we have presented a review of a small selection of entrenched assumptions that have stymied intelligence theorizing. In doing so, the central point of our argument is that we have focused for too long on between-individual comparisons and too willingly tolerated inconsideration of within-person accounts.

Psychometric tests of intelligence have great utility in predicting interesting (and important) outcomes, and pragmatically the common-factor analyses of correlations works well in this regard. One might be tempted to therefore ask, what are the implications of not redressing the limitations reviewed in Part 1? This is our response so far. First, if we do not question the supposition of stability, we risk over-looking (and not assessing) adaptive, situation-contingent, within-person differences. This risks limiting our understanding of the dynamic features of intelligent behaviour in applied settings, such as work and education. Second, we reviewed analyses that demonstrate assuming within-person accounts follow from between-person theories, that is, assuming ergodicity, is logically untenable. The ergodic claim assumes stationarity of means and covariances across time within the individual, and this is largely untenable in practice, further contributing to the argument for testing the supposition of stability. Third, we reminded readers that the between-person theories themselves are often based on an untested assumption of reflective models, that differences in the indicators are caused by differences in the latent variable (arrows going from the latent variable to the indicators). The alternative, formative claim, that indicators are causing differences in the latent variable (arrows going to the latent variable from the indicators) is rarely tested, and when it is, the respective models often account for as much variance as reflective models, so the choice can easily be driven by pragmatism and inertia. 

When we scratch the surface, it is apparent that between-person models of intellect have little explanatory value and thus their pragmatic benefit and descriptive utility rests on a theoretically shallow house of cards. To address the challenges presented by these and other types of entrenched assumptions, we need a grounded process theory of intelligence. In the following we map out some of the requirements needed for a within-person approach, admittedly in somewhat of a selective way.

## 3. Part 2: Requirements for A Within-Person Approach to Intelligence

“*It is true that the components of individual differences have often been interpreted in terms of cognitive processes, but such an interpretation does not logically follow. The interpretation is necessarily a post hoc interpretation based on the assumptions that processes are directly reflected in individual differences in performances and that correlation between performances defining a factor indicates that a common process is involved.*”

(De Boeck et al. 2020, p. 58)

### 3.1. Process-Oriented Accounts

Following the arguments of Molenaar (2004, 2013) and Borsboom et al. (2003, 2004), the ergodic assumption in psychology is tenuous at best, and all between-person models are variously imperfect accounts of what is likely to be occurring within an individual. Taking the call for the study of within-individual variability in its own right seriously (Molenaar 2004), where does one begin to map out a process-oriented account? The obvious choice is with working memory, and we will consider what current conceptualisations of working-memory theory have to offer. However, it turns out the notion of complexity is a compelling first place to start because of its already deep links with intelligence theory. 

#### 3.1.1. Complexity as the “Ingredient” Process of Intelligence

Theorising within the psychometric intelligence tradition is not completely devoid of attempts to understand processes. Arguably the most developed is based on the notion of *complexity*, and the observation that performances on tasks, occupations, and work that are more complex, broadly defined, tend to be more highly correlated with intelligence. The ensuing supposition is that intelligence entails a capacity to deal with complexity (Gottfredson 2018). Following from this, an independent indicator of complexity is changes in correlations with, or loadings on, measures of intelligence that are concomitant with changes in task complexity (Arend et al. 2003; Spilsbury et al. 1990; Stankov and Cregan 1993), but all else being equal, not with changes in difficulty generated by other task features (Birney and Bowman 2009; Stankov 2000). Birney (2002) referred to this criterion as *psychometric complexity*.

#### Complexity vs. Difficulty

To understand why complexity is of such value in the conceptualisation and assessment of intelligence, it is necessary to take a brief diversion to distinguish it from difficulty (Beckmann et al. 2017). Difficulty is atheoretical, in that a rank-ordering of test items that are solved by fewer and fewer people tells us little about what make items difficult, just as correlations alone, we will argue, tell us little about complexity. *Difficulty* is a statistical concept captured by indices such as the proportion of people who answer an intelligence test item correctly. Complexity is a *cause* for the difficulty one experiences, in that it is a consequence of the cognitive processes demanded of the task at hand.

While *complexity* is often equated to difficulty, there are certainly tasks that are not difficult yet predictive of intelligence. For instance, the well-known, perceptual *inspection time* task (Deary 2001) appears to impose minimal storage or processing load, yet is a good predictor of *Gf*. Similarly, performance on the relational monitoring task (Bateman et al. 2019; Chuderski 2014) is highly predictive of *Gf*, but the reasoning and memory demands are ostensibly minimal. Complexity is more nuanced and entails systematic manipulations based on a structural process hypothesis regarding differential demand on ability (Lohman and Ippel 1993). That is, complexity is a causal-formative concept that is indexed by performance across task manipulations that have conceptual unity. It is conceptualised first and foremost as a quality that is determined by the cognitive demands that characteristics of the task and the situation impose, and because of this, it is psychologically substantive. Accordingly, manipulations monotonically ordered by complexity are manipulations of monotonically increasing demand on the psychological attribute (Birney et al. 2019). Only in a truly pure, unidimensional task will the complexity continuum coincide with the difficulty continuum. Of course, such tasks do not exist. However, with careful, theory-driven task analyses, the parameters of complexity can be formalised and investigated (Beckmann 2010; Birney and Bowman 2009; Ecker et al. 2010; Goecke et al. 2021; Halford et al. 1998).

Differential complexity correlations are a plausible, necessary criterion of an increase in cognitive demand. However, there are some statistical and theoretical challenges to be flagged. Statistically, by definition, the magnitude of a correlation coefficient is influenced by the upper-bound variance of their component measures, and variances in ability tasks are influenced by statistical difficulty. Due to restrictions of range, all else equal, tasks that are of average statistical difficulty will have a higher upper-bound variance than both easier or more difficult tasks, attenuating correlations in both the latter cases. In practice, easier and harder tasks may appear “less” complex than they really are. Whether the “shrinkage” of random-effects in multilevel models (which we describe in Part 4) serves to bring extreme observations toward the fixed-effect (i.e., toward the mean intercept or slope), or the “task purification” of latent variable SEM models are useful ways to address this statistical limitation needs further investigation.

Theoretically, once again, appropriateness of complexity correlations assume we have a sufficiently detailed process-account of the latent attribute to inform a causal statement of how the complexity manipulation demands a concomitant investment of concordant intellectual processes (Sternberg 1977b, 1980). That is, while we have a theoretical cause (complexity) and a way to assess its effect (correlations), alone it provides little understanding of antecedents—anything that leads to increased correlations with intelligence is presumably a complexity manipulation. In response to this ambiguity, an early approach to incorporate theory was to consider performance under competing task conditions (Fogarty and Stankov 1982) or by increasing the number of mental permutations required to successfully solve a set of reasoning tasks (e.g., Schweizer 1996; Schweizer and Koch 2002; Stankov 2000; Stankov and Crawford 1993). Such manipulations were shown to also lead to increases in correlations with *Gf*, and hence was presented as further evidence of the importance of complexity.

Birney et al. (2019) defined *psychometric complexity* more formally and generally as the extent to which *within-individual* differences in task performance across theoretically substantive complexity manipulations differ as a function of *between-individual* differences in that attribute. In multilevel models, this is a cross-level interaction. That this is the case, explicates a possible conceptual definition, operationalisation, and assessment of intelligence as cognitive flexibility that is formally aligned and testable within a common methodological framework. We discuss this further in Part 4.

#### 3.1.2. Working-Memory Accounts of Intelligence

Investigations of processes in individual differences research has had a strong focus on understanding mechanisms underlying working memory (WM) in and of itself (e.g., Ecker et al. 2010; Goecke et al. 2021; Oberauer and Lewandowsky 2016), or as a set of processes common to both WM and *Gf* (e.g., Ackerman et al. 2005; Engle et al. 1999; Oberauer et al. 2007; Shipstead et al. 2016). What is common in many of the studies and approaches described in the rest of this section is the combined experimental-correlational methodology—basic processes are proposed, operationalised as individual differences variables and “measured”, and then “validated” as incremental predictors of the latent attribute (e.g., WMC or *Gf*). The latent variables representing these attributes are defined and operationalised using the traditional reflective procedures we have described. The supposition is that the more variance the proposed processes predict in the latent WM or *Gf* variable, the more we know about working memory or intelligence. The view we advocate is that this approach, while rightminded in explicating process accounts, is incomplete.

In terms of WM-focused studies, consider for instance Ecker et al. (2010), who sought to map processes underlying working memory updating. Following a task analysis of a set of commonly used updating tasks, they identified three component processes, retrieval, transformation, and substitution. Using a modified version of the memory updating task, they manipulated the absence or presence of each component experimentally, and used multilevel, mixed-effects modelling to test theoretically specified contrast hypotheses (this is similar to the costs approach used by Bateman and Birney (2019) to identify a link between relational integration demand and *Gf*, which we will describe shortly). Ecker et al. first demonstrated that the WM updating components were distinct and additive in predicting task response times and accuracy (there were no observed interactions between the components). In the second part of the Ecker et al. study, a bi-factor SEM model tested and confirmed differential associations of the three WM updating components with an independently defined (reflective) latent WMC factor.

In a recent study investigating the role of the working-memory binding hypothesis, Goecke et al. (2021) combined an experimental manipulation of complexity of elementary cognitive tasks (ECTs), also using a bi-factor SEM approach to identify the mechanisms underlying binding demands (e.g., more stimulus-response mappings = greater binding demand) on working memory capacity. This was achieved in a three-step process. First, given ECT performance is typically differentiated more by response latency rather than accuracy, performance indices were derived using drift diffusion modelling. In total, standardized drift rates were derived for 12 indicators, 3 speed tasks (change-detection, stimulus comparison, substitution) by 2 modalities (selected from either letter, figure, or number modality) by two binding complexity levels (low and high). Second, a bi-factor SEM was run where all 12 indicators were freely allowed to load on a *general* speed factor, and only the six high complexity binding indicators defined the *specific* binding factor. Third, these two process factors were then regressed on an independently derived WMC latent factor. The results suggest that both the general and high-binding factors were comparable and significant unique predictors of WMC, together explaining 66.5% of the variance in the latent WMC factor.

In terms of combined *Gf* and WM studies, Unsworth and Engle (2007b) for instance reported a complexity effect with *Gf* in simple-span tasks using a combined experimental/individual-differences approach. The authors demonstrated that as the number of to-be-recalled elements increases in simple-span memory tasks to supra-span levels, determinants of performance become more like complex-span WM tasks, in that there was an emergence of a monotonic increase in correlations with *Gf* as a function of list-length. Shipstead et al. (2016), building on this and other extensive theorising (e.g., Engle 2002; Engle et al. 1999; Unsworth and Engle 2007a), proposed that the link between WM and *Gf* has to do with the engagement of executive attention for maintenance and disengagement processes of information held in the focus of attention. Importantly in the Shipstead et al. (2016) conceptualisation, these executive processes do not simply covary with *Gf*, but rather are ontological to both *WM* and *Gf.* This is such that *Gf* and WM tasks require executive attention of both maintenance and disengagement, but to different degrees. They argue disengagement is more critical to *Gf* tasks, whereas maintenance is more critical for WM tasks. Additional work has investigated a range of different WM tasks and their relations to *Gf*, such as inhibition of lure trials in the updating n-back task (Burgess et al. 2011; Gray et al. 2003).

While WM processes are important aspects in *Gf* tasks, they are not the only aspects important to intelligence. For instance, Sternberg (1977a) identified encoding, mapping, and application processes (“components” in his parlance) underlying analogical reasoning. From a task analysis perspective, understanding reasoning and novelty processing is also important, and theories of complexity in terms of *processing* capacity limits (e.g., Halford et al. 1998) are well positioned to progress further investigations (Birney and Bowman 2009).

#### 3.1.3. Relational Binding and Integration Accounts of Intelligence

One way of thinking about how *processing capacity* limits are related to complexity is in terms of relational binding and relational integration demand. Oberauer and colleagues (e.g., Oberauer 2021; Oberauer et al. 2000) suggest a set of working-memory mechanisms by which a coordinate system binds relational information between *content* (say, for instance, a mountain and mole hill) and *contextual* information (a size comparison) to facilitate action on a specific mental representation to derive a response (e.g., the mountain is larger). Limitation on accessibility of chunks is determined by constraints on the capacity of the *focus of attention* and priming in the *region of direct access* (Oberauer 2013). 

Relational integration and precursor processes associated with relational binding are also thought to underly the associations between *WM* and *Gf.* We have used relational complexity (RC) theory to parameterise the cognitive demand of *relational integration* (Bateman and Birney 2019; Bateman et al. 2019; Birney and Bowman 2009; Birney et al. 2012; Gabales and Birney 2011). RC theory is based on the premise that the limits of *WM* can be understood in terms of the complexity of to-be-instantiated relations (Birney and Halford 2002; Halford and Wilson 1980; Halford et al. 1998; Halford et al. 2010). A binary relation entails two arguments, as in the relational concept: LARGER-THAN(mountain, mole hill). A relation is instantiated through the binding of a value to an argument-slot, such as “mountain” to the larger-than argument; and separately “mole hill” to the implied smaller-than argument-slot. The relation exists only in its integrated form. It is thought that the typical limit of human capacity is a quaternary relation, an example of which according to Halford et al. (2007), are proportional analogies in the form of A:B :: C:?. 

Application of RC theory led to the development and validation of a class of relational integration measures known as Latin Square Tasks (LST) (Birney et al. 2006). A Latin Square entails a k × k matrix with k different element types distributed such that each element exists only once in each row and column. Experimental manipulations of partially completed LS are in terms of (a) relational complexity (relational integration of 2, 3, or 4 dimensions) and storage load (number of interim solutions to be maintained) (Birney and Bowman 2009; Birney et al. 2006); (b) presentation format (with and without time-limits) (Hearne et al. 2019) (c) *dynamic-completion* (recording of non-target-cells as external-memory aid to mitigate memory demand and isolate binding) (Bateman et al. 2017); and LST dimensionality (4 × 4 LST, requiring only a shape response, and a 5 × 5 Greco-LST which superimposes two LSTs integrating shape and colour) (Birney et al. 2012; Gabales and Birney 2011). Each of these within-task manipulations were theoretically designed to tap specific aspects of *Gf*; they have been shown to be differentially and incrementally predictive to varying extents. 

RC has also been useful to inform manipulations of relational binding in cognitive processing load in the Arithmetic Chain Task (ACT) (Bateman and Birney 2019) and the Swaps task (Bateman 2020; Stankov 2000), where systematicity plays out differently in each, giving further insights into underlying within-individual mechanisms. For each trial in the experimental conditions of the ACT, participants are given 6s to study a to-be-recalled mapping of letters to numbers (Screen1: A = 2, B = 4, C = 1). They are then given new mappings that are either in a *systematic* order (Screen2: X = A, Y = B, Z = C) or a *random* order (e.g., X = B, Y = C, Z = A), and need to use this derived mapping of numbers on to X, Y and Z to complete a chain of simple arithmetic (Screen3:, e.g., 5 − 4 + X + 2 − Y + Z = ?). Systematicity inherent in natural-ordering facilitates chunking of relationally bound elements (ABC = 241 = XYZ), which aids number recall to complete the arithmetic. Random (or non-systematic) ordering stymies chunking (ABC = 241 = ZXY). Using multilevel models, the within-individual *cost* of performance in the *non-systematic* condition (relative to a control condition with no mappings) was shown to be moderated by *Gf*, but not for the systematic condition (Bateman and Birney 2019). The interpretation is that sensitivity to systematicity and capacity to build strong flexible bindings in disordered contexts (ABC = 241 = ZXY) is an important *Gf* process.

The Swaps task requires mental permutation and updating and presents participants with a letter triplet (e.g., JKL) with instructions to mentally rearrange or ‘swap’ the positions of letters (e.g., Swap 1 and 2; then Swap 3 and 2) and report the final ordering (i.e., KLJ). As indicated previously, Stankov and Cregan (1993) have demonstrated the greater the number of mental permutations the higher the correlation with *Gf*. Bateman (2020) modified the Swaps task to target binding systematicity designed to emerge over the multiple swaps required within items. For example, given [TQXBL] the required solution path with swap instructions is: Initial order [TQXBL]; Swap 1 with 2 = [QTXBL]; swap 3 with 2 = [QXTBL]; swap 1 with 3 = final order [TXQBL]. The intended systematicity is that B and L can be chunked because they are never swapped and this is not pointed out to participants; and *sensitivity* to this facilitates performance. Based on the ACT findings of Bateman and Birney (2019), one might predict that performance in the intuitively more difficult, non-systematic condition would be more predictive of *Gf*. However, preliminary data provided by Bateman (2020) indicated the opposite—performance was moderated by *Gf* when *systematicity was present*, but not when it was absent. This suggests that sensitivity to systematicity over time is also a feature of *Gf*.

As a relevant aside, the notion of fluid intelligence comprising the ability to utilise structure (where and when available) in conjunction with the result of poorer performance in the non-systematic condition resonates with findings in relation to the so-called semanticity effect in complex problem solving (Beckmann 1994; Beckmann et al. 2017; Beckmann and Goode 2013). Here, the presence of semantically laden labels for system variables negatively affects knowledge acquisition as well as system control performance. This effect is caused by relying on a false sense of familiarity which is triggered by the variable labels rather than systematically testing assumptions. In other words, the apparent lack of systematicity when interacting with the system results in not utilising available cognitive resources, which is reflected in lower correlations between *Gf* and CPS performance shown under high semanticity conditions in contrast to CPS performance shown under low semanticity conditions.

Together, the ACT and the Swaps data support conceptual definitions of *Gf* as entailing both a capacity for binding sensitivity to systematicity and managing disorder through building and maintaining strong yet flexible bindings. The standard between-person approach tells us that both tasks correlate with *Gf* to the same extent (*r* ~ 0.40); the within-individual approach provides additional insights by suggesting they do so for different reasons, supporting our argument that understanding within-individual processes is critical to intelligence as cognitive flexibility.

### 3.2. Summary of Part 2: Why WM Theory Is Important to Within-Person Process Accounts

In Part 2, we outlined the historical importance of the concept of “complexity” in intelligence theorising and made a distinction between difficulty as a statistical entity and complexity as a theoretical concept. While there are pragmatic challenges in operationalising this distinction, we alluded to the promise of MLM, when clearly specified process accounts are incorporated into the operationalisation. In this respect, we reviewed seminal process accounts of WM in relation to fluid intelligence, and more recent advances in terms of the cognitive models that formalise the role of relational binding and integration. In particular, we highlighted exemplar research that has incorporated process-accounts in SEM modelling (e.g., bi-factor analyses). The core point is that because of the limitations outlined in Part 1, process accounts are needed for any theory that wishes to take within-individual differences seriously. In our view, the process accounts reviewed in this section provide an excellent place to start.

## 4. Part 3: Theory through Task Analysis

While the work so far presented certainly takes a process account, there are two issues left unaddressed. First, the tasks investigated are not dynamic and nor do they necessarily allow for within-task adaptation to changing conditions. Second, the “validity” criterion used are predominantly non-dynamic measures of WM and *Gf*. To validate an operationalisation of intelligence as cognitive flexibility in a traditional way (i.e., through statistical associations), one needs an *appropriate* dynamic criterion measure. The standard approach would be to predict a real-world outcome where “cognitive flexibility” is assumed to be required, and to then check for incremental prediction of this outcome over and above classic measures of *Gf*. This is the approach used for validating CPS tasks, and other “alternative” measures of intelligence. This seems conceptually the right thing to do, however defining what is appropriate is not straightforward, although the necessary steps are clear. First, one must resist the pragmatics of relying on readily available quantified criteria (i.e., statistical association) without reflection on their conceptual and operational quality. If one relies on such atheoretical approaches there are two possible outcomes: (1) there is a correlation of some size and we happily conclude we have valid “measurement”, or (2) there is no (or unsatisfactory) correlation, and conclude the criterion was not good enough, but that our “measurement” might be saved from negative evidence while we search for the right criterion. A more systematic approach is needed. In response to these sort of challenges, we begin by distinguishing between features and dimensions that differentiate static vs. dynamic tasks, and consider how the former might be modified to emulate the latter.

### 4.1. Static Tasks

Static assessment tasks have several common characteristics. They (a) focus on the accuracy or speed of a *one-off* response; (b) follow classic psychometric principles closely, particularly the notion of item stability as the foundation of measurement consistency and test development; (c) assume local independence of items, whereby items are ostensibly *interchangeable* (Pedhazuer and Schmelkin 1991), and (d) item-specific feedback is not provided (as this would jeopardise (b) and (c)). Due to these properties, performance in static tests is typically operationalised as an aggregate of item accuracy (e.g., proportion of correct items) or response time. Whilst static tasks may be psychometrically desirable, they are conceptually inadequate when it comes to dynamic concepts such as intelligence as cognitive flexibility. Static tasks can be made dynamic by focussing on the variability (in accuracy/speed) caused by systematic within-task manipulations. This can be achieved in a number of ways, we discuss two general approaches that entail (a) redesigning tasks to entail structured within-task manipulations, and (b) through interposition of idiosyncratic information to the existing task.

#### 4.1.1. Theoretically Substantiated Within-Task Manipulation 

When items are designed to be differentially sensitive to the structure of specific underlying cognitive processes, they are fundamental and not interchangeable in relation to items of a specifically, different type. Performance is conceptualised as a function of this predefined structural relationship, the simplest being a relation of difference. This is a standard approach for identifying processes as we have already outlined (e.g., Ecker et al. 2010). One’s capacity to learn can also be modelled as changes in performance from one item to the next in linear and non-linear ways, controlling for other task and person characteristics—that is, item-order is the relational structure. Using an MLM approach, Birney et al. (2017) investigated correlates of performance and item-order experience trajectories across the 36 items of Raven’s Advanced Progressive Matrices test. Similar approaches to item-order effects have been conducted by Schweizer and colleagues (e.g., Schweizer 2009; Schweizer et al. 2015). The relational structure can also be variable and nuanced. For instance, using Bayesian methods, Cripps et al. (2016) separately and jointly modelled the probability of an individual to spiral monotonically into poorer performance during a natural decision-making task, which are sometimes referred to as microworlds, if and when they reached an idiosyncratic *motivational* threshold (as opposed to an ability threshold). Birney et al. (2021) report on preliminary work extending Cripps et al.’s to model spiral and recovery trajectories in the n-back task.

#### 4.1.2. Within-Task “Interposition”

Static tests can also be made more dynamic through interposition of information during a task that intentionally serves to focus problem solving on one or more item characteristics. This can be in the form of feedback, such as simple accuracy feedback, or a more specific strategy/hint, such as “consider how colours change” in a series-completion task. Provision of feedback designed to change performance is one of the defining features of the *dynamic testing* paradigm (Guthke and Beckmann 2000), but other forms of prompting may also change the way people approach problems. While the intention of such manipulations is to focus assessment on dynamic processes rather than static ones, an important theoretical implication of interpositions is that they may impact the validity of the assessment in unintended ways (Birney et al. 2022; Double and Birney 2019). Careful theorising and experimentation are necessary to ensure validity claims can be defended. Our approach is to base interposition manipulations on a process account of intelligence as cognitive flexibility.

### 4.2. Dynamic Tasks

The main characteristic of dynamic tasks—as they have been employed in the context of complex problem-solving research and the assessment of learning ability—is their operational focus on within-person performance variability. The definition of Dynamic Testing, for instance, characterises it as a methodological approach to psychometric assessment that uses systematic variations of task characteristics or situational characteristics in the presentation of test items with the intention to evoke intra-individual variability in test performance (Beckmann 2014; Elliott et al. 2018; Guthke and Beckmann 2000). In so-called learning tests the dynamic nature of assessment is realised by providing test takers with the opportunity to demonstrate their receptiveness to scaffolded, error-specific thinking prompts after an incorrect response to a test item. Complex problem solving can also be conceptualised as dynamic testing (Beckmann 2014) as it also embodies various forms of dynamics. These include (a) the feature of system feedback (e.g., whether the system state changes towards the set goal state as a consequence of the problem solver’s intervention), (b) the implementation of so-called autonomic changes in the system behaviour (i.e., the state of system variables changes independently from the problem solvers inputs), but also (c) the necessity for knowledge-acquisition (rule-learning) on which subsequent system control (rule application) relies (Goode and Beckmann 2010).

In short, dynamic tasks have two or more dimensions of performance, entail fluid and divergent processes, and are multi-phasic (rather than multi-dimensional) across time/occasion and across the external (task context) and internal (cognitive process) problem-space. Dynamic processes are present to some extent in existing flexibility and switching tasks (Miyake and Friedman 2012), but as we have just outlined, are arguably better represented in complex problem solving (CPS) and microworld tasks (Dörner and Funke 2017; Funke et al. 2017), which as also argued above, may have a formative nature as complex-problem solving competencies. We consider each of these paradigms next.

#### 4.2.1. Set-Switching and Card Sorting

The well-known set-switching paradigm entails learning and applying a set of conditional rules. For instance, the screen location of a stimulus (left/right) might be associated with a Y/N response conditional on a particular stimulus feature (colour/shape), for example: “Y if stimulus is on left and green, else N; Y if stimulus is on right and circle, else N”. Performance requires rule-set acquisition, conditional response-switching, and inhibition (e.g., not pressing Y when a green square is on the right). Performance is a function of a response-latency *cost* for switch trials relative to repeat trials. While the basic cognitive psychology switching research tends not to consider individual differences (cf., Ravizza and Carter 2008), it has been useful as a metaphor of higher level shifting of perspectives, as might be necessary in novelty processing (Beckmann 2014; Diamond 2013), or as formative indicators for higher level flexibility concepts. The *Wisconsin Card Sorting Task* requires one to sort cards one at a time based on a core attribute (colour, shape, numerosity). Unlike set-switching, the sorting rule is not known in advance, rather it needs to be deduced from feedback. This rule (say, sort by colour) will persist across multiple trials and then *change without forewarning* to a different rule (say, sort by shape). *Preservative* sorting in the face of negative feedback indicates a lack of cognitive flexibility. Recent computational modelling research has shown the diagnostic value of deriving alternative assessment metrics from well-known neuropsychological tasks, such as these. For instance, Steinke and Kopp (2020) demonstrated that a reconceptualisation of Wisconsin Card Sorting Test metrics show promise in clinically differentiating Parkinson and ALS conditions. It is important to note that while parameterizing task performance using computational methods can lead to effective prediction/diagnosis, it is not given they will also lead to sufficient theoretical understanding necessary to design interventions.

#### 4.2.2. Complex Problem Solving (CPS) and Microworlds

CPS tasks present participants with an explicit opportunity to acquire knowledge and to control and manage changes in a complex system by allowing direct experimentation (Dörner and Funke 2017; Funke 1998). CPS tasks vary from high-fidelity *microworld* simulations with many inputs and outputs (e.g., flight simulators), to “*minimal complex systems*” (MCS) which present the simplest possible interaction of variables (ie, deterministic and linear) (Funke et al. 2017). CPS tasks having conceptual links with intelligence and decades of successful application in training and education (Wood et al. 2009). However, they are often discounted as intelligence measures because of the challenge in extracting psychometrically reliable and valid performance indicators *that correlate sufficiently with static tests of intelligence* (Beckmann and Guthke 1995; Greiff et al. 2015; Stadler et al. 2015). Consistent with others (Funke et al. 2017), we argue that emphasis on classic psychometric qualities has led to an advocacy for *MCS-like* tasks, a reduction in multi-phasic task complexity, and questionable validity as tests of “true” CPS ability (Beckmann et al. 2017). As indicated previously, it is feasible that typical summary scores from CPS represent a composite-formative concept, and according to Bollen and Diamantopoulos (2017) are not measures. This is not necessarily an insurmountable problem. We have argued that a sufficiently detailed task analysis and experimental manipulations, causal and effect-based concepts can be specified and extracted as measures (Beckmann 2019; Birney et al. 2018).

### 4.3. Summary of Part 3: Why Task-Analysis Is Important 

A tacit “known” we have not previously mentioned is the mantra that one should “validate” new measures of intelligence by assessing how well they correlate with existing ones. This not only leads to new tests functioning much like old ones, but also results in theoretical inertia; our understanding of intelligence and how to measure it does not progress as rapidly as it could. To bring operationalisations of intelligence in line with conceptualisations, we must stretch beyond the status quo (which we have outlined in Parts 1 and 2). With this as our overarching goal, in Part 3 we reviewed features common to existing static and dynamic assessment tasks. We surmised that static tasks are, inter-alia, characterised by one-off measures and local independence of items, whereas dynamic tasks are characterised by having multiple dimensions of performance across items that have dependences across multiple occasions, and often entail feedback. The latter is conceptual closer to our proposed within-individual conceptualisation of intelligence, however, as we pointed out, dynamic tasks present challenges to standard psychometric methods that seems to have reinforced pragmatism and inaction. In the next section we describe how multilevel models (also known as latent-growth models) can address these challenges. 

## 5. Part 4: A Case for Multilevel Models in Intelligence Research 

As we have suggested above, with careful, theory-driven task analyses, the parameters of complexity can be formalised and investigated (Birney and Bowman 2009; Ecker et al. 2010; Goecke et al. 2021; Halford et al. 1998). Multilevel models (MLM) are well-suited for this in that they provide a means to explicate a definition, operationalisation, and assessment of cognitive flexibility that is formally aligned and testable within a common model. Such formalisations facilitate statistical analyses, but are also a priori critical for theoretical developments (Navarro 2021). The goal of this final section is to explain how MLM might be used as a theoretical framework for intelligence as cognitive flexibility.

### 5.1. Cognitive Flexibility as Contingent Level 1 Variability in MLM Models

In considering a within-person account of intelligence, there are a number of sources of variability to consider. Variability at the level of the sample (as a proxy for the population, i.e., Level 2 between individuals), variability at the level of the individual (Level 1, within-individual), and cross-level variability. These can be represented as random effects in a multilevel model. An example of a regression approach is represented below, although SEM formalisations are of course comparable (Brose et al. 2021). 

Level 1:

(1) $$Y_{ij}=\pi_{0j}+\pi_{1j}\cdot X_{ij}+\pi_{2j}\cdot Z_{i}+e_{ij}$$

Level 2:

(2) $$\pi_{0j}=\beta_{00}+\beta_{01}\cdot W_{j}+\beta_{02}\cdot V_{j}+r_{0j}$$

(3) $$\pi_{1j}=\beta_{10}+\beta_{11}\cdot W_{j}+r_{1j}$$

(4) $$\pi_{2j}=\beta_{20}+\beta_{21}\cdot W_{j}+r_{2j}$$

where, $Y_{ij}$ = observation *i* for individual *j*.

In this two-level model, *π*_0*j*_ represents the mean score (i.e., an intercept) for individual *j* across all occasions *i* (when *X* and *Z* are centred); whereas *π*_1*j*_ and *π*_2*j*_ represent the change in *Y*, as a function of *X* and *Z, respectively*, also observed at level 1 (i.e., slopes). Here, we make a distinction between two different types of level 1 variables, *X* and *Z*. *X* is a variable that varies by occasion (i) *and* individual (j), such as a participant’s rating of confidence or perceived task demand for the given occasion, hence the subscripting, *X_ij_*. *Z* on the other hand, is a variable that changes by occasion (i) only; it is constant for all individuals for that occasion and accordingly subscripted as *Z_i_*. An example is an item feature, such as item complexity manipulation, presented in a constant order for everyone, or a variable such as time. While in practice these variables are typically treated as equivalent statistically, in terms of cognitive flexibility they are conceptually different. The model could be extended (with subscripts updated) to capture person × task × situation interactions (Beckmann 2010) by adding a clustering level, such that we have observation *Y_ijk_*, where individual *i* (now at level 3) under situation *j* (*Z*, now at level 2) attempts task manipulation *k* (*X*, now at level 1), but for illustrative purposes we stay with the two-level conceptualisation.

Variability in the individuals’ *π*_0*j*_, *π*_1*j*_, and *π*_2*j*_ parameters is considered at level 2 (in Equations (2)–(4), respectively). *β*_01_ represents the change in the individuals’ mean scores as a function of *W*, a variable that differs between people; and *β*_11_ and *β*_21_, respectively represent the change in the individuals’ *X* and *Z* slope parameters, also as a function of W. Accordingly, *β*_11_ and *β*_21_ are cross-level interaction parameters. For completeness, *β*_00_, *β*_10_, and *β*_20_ represent the sample’s average mean and slope (conditional on level 1 and level 2 variables). One might also be tempted to make a distinction between types of level 2 variables analogous to that made between *X* and *Z*. For instance, *W* might reflect inherent individual differences, such as age or conscientiousness, whereas *V* (Equation (2)) might represent a factor external to the individual, such as a between-condition manipulation (e.g., group 1 gets contextualised feedback, and group 2 gets generic feedback). While the latter is of potential scientific interest and allows for experimental group comparisons for the purpose of, say, validating an operationalisation of cognitive flexibility, our focus here is specifically on within-person processes and how they might differ from one person to another. Accordingly, this type of between-condition comparison is not a factor directly of relevance in building a conceptualisation and measure of cognitive flexibility.

#### 5.1.1. Within- and Between-Individual Parameters of Intelligence as Flexibility

We postulate that cognitive flexibility can be conceived as level 1 variability in (intellectual) behaviour (*Y_ij_*) that has level 1 contingency. That is, as a behavioural response to *X* and *Z* factors as just described. *Π*_1_ and *π*_2_ are contingency parameters, potentially conditional on level 2 influences. The contingency parameters represent how one’s responses change as a function of variation in the problem-space (broadly defined in terms of *X* and *Z* factors). *X* and *Z* are exemplar triggers in the problem-space for a dynamic response. The magnitude of such responses is indexed by the contingency parameters, and these might be moderated by specific characteristics of the individual. For instance, someone already predisposed to novelty (such as someone high in the openness personality dimension) may not require an as extreme contingent response as someone low in openness; their higher levels of openness might mitigate the flexibility needed when confronted with *X* and *Z* factors. This between-person moderation of level 1 contingencies is represented by W parameters, specifically in our representation by *β*_11_ and *β*_21_. The *β*_0_ intercept parameters reflect group/population mean levels of the contingency parameters. However, simply because the *β*s are between-person parameters, this does not mean they are not relevant to a conceptualisation of within-person flexibility. The moderation effect just described, demonstrates that these between-person parameters are critical because they serve to contextualise individual responses, the *Y_ij_*, more fully. Table 1 presents a selection of possibly relevant level 1 contingent factors and level 2 moderators of these.

The contingent variables can be conceived as either person-centred (*X*) or task/situation centred (*Z*), although each idiosyncratically impact the person’s response. The *X* factors are contemporaneous to the response in some way, but conceptually distinct from it. For instance, confidence in accuracy ratings are retrospective to a response, whereas state personality is antecedent to a response, but in both cases, they are distinct and idiosyncratically experienced by the individual. On the other hand, the *Z* factors are germane to the required response, and while they might differ from one occasion to another, they are objectively the same for all people, such as the binding complexity of an item. People are likely to differ in their response to the complexity (i.e., between-individual differences), and this variability is captured in the random-effects of the respective *π* contingency parameter.

#### 5.1.2. Statistical Advantages of MLM

Multilevel models are considered to resolve reliability concerns about using difference scores (Draheim et al. 2021), allowing contrasts between conditions of, say, higher vs. lower complexity (Birney et al. 2017; Conway et al. 2021; Frischkorn and von Bastian 2021). There are also other methodological concerns related to using correlation-based criteria that MLM is well positioned to address. Low complexity tends to be associated with higher accuracy (indicating lower levels of experienced difficulty) and a small number of potential solution paths, which by definition lead to ceiling effects and consequently to lower reliability. Higher complexity items tend to have lower accuracy, and a larger number of potential (and perceived) solution paths, which might introduce a combination of floor effects and multidimensionality^6^, also resulting in lower reliability. Having the basis for the correlation-criterion of psychometric complexity to “work” across more than a small range of complexity levels is challenging, particularly since the extremes often define the scope of interest. Within LMER models, shrinkage of random-effects toward fixed-effects (Gelman et al. 2012) has the potential to address this to some extent, although more research is needed to understand the boundaries. An alternative approach is to adopt a binary perspective, where the process is required (present) or not (absent). Ecker et al. (2010), Bateman and Birney (2019), and Birney et al. (2018) have each used this effectively under different conditions. 

### 5.2. Microworld Contingency Parameters as Indicators of Cognitive Flexibility: A Case Study

**Figure 3:**
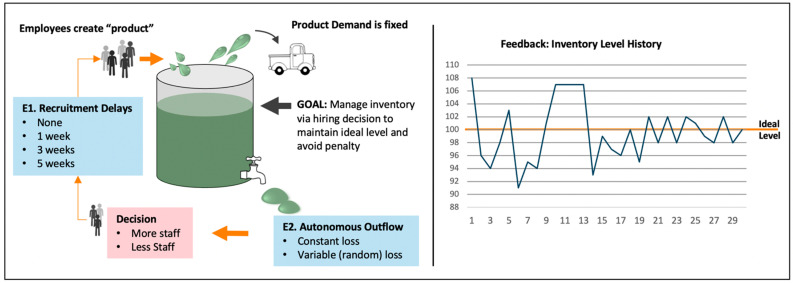
Schematic representation of microworld task described by Birney et al. (2018) and experimental manipulations (E1 and E2) with exemplar trial-by-trial inventory level feedback across 30 decision periods (which defined a single “attempt”).

Using multilevel models, our previous work (Birney et al. 2018) suggests that judicious manipulations of microworld parameters offer potential to derive indicators of decisional and reasoning processes underlying intelligence, that can be isolated from other factors. Although the study was not designed to operationalise intelligence as cognitive flexibility in the way we conceive of it here, the LMER application of parameters derived from this work exemplifies our current approach. In this study, participants were tasked with maintaining a dynamic (changing) inventory at an ideal level by managing outflow via staffing decisions over 30 simulated weeks (see Figure 3).

Complexity was experimentally manipulated along two independent dimensions intrinsic to solution, *delays* and *outflow* (these would be *Z* factors in Table 1). Delays (Figure 3, E1) occurred with regard to hiring and firing staff and have a knowable fixed, relational structure. A greater delay between decisions and their impact was expected to generate a concomitant increase in working memory demand. Outflow (Figure 3, E2) was either constant or variable (random). Variable *outflow* resulted in less predictable deviations from the ideal inventory level than when outflow was at a constant rate. Due to the inherent uncertainty, variable outflow was expected to make the task difficult to manage. However, for the same reasons (i.e., uncertainty), reasoning ability was expected to be less effective in mitigating this type of challenge, although we argued that there may be some strategies that might help, given sufficient motivation to attend to detail. Dynamic trial-by-trial feedback across a given block was presented to participants in graphical format (e.g., Figure 3, right panel). The penalty score analysed as the dependent variable was calculated as a function of the trial-by-trial discrepancies between the impact of participants decisions and the ideal inventory level accumulated by the end of the block. Participants had multiple attempts under different delay and outflow conditions, and therefore experience (attempt number) was an additional performance parameter (which would also be a Z factor in Table 1).

**Figure 4:**
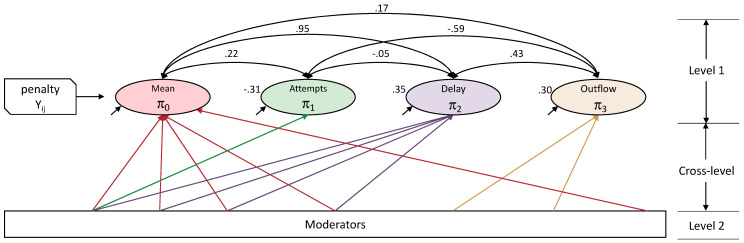
Intercorrelations and graphical representation of fixed-effects from MLM analysis of microworld performance indexed by accumulated block penalty (adapted from Birney et al. 2018, with permission from Elsevier; ref: 5356931314753). The model was of the following general form: Level 1: [$Y_{ij}=\pi_{0j}+\pi_{1j}\cdot Attempt_{j}+\pi_{2j}\cdot Delay_{i}+\pi_{3j}\cdot Outflow_{i}+e_{ij}$]; Level 2: [$\pi_{0j}=\beta_{00}+\beta_{01}\cdot Moderator_{j}+r_{0j}$]; [$\pi_{1j}=\beta_{10}+\beta_{11}\cdot Moderator_{j}+r_{1j}$]; [$\pi_{2j}=\beta_{20}+\beta_{21}\cdot Moderator_{j}+r_{2j}$]; [$\pi_{3j}=\beta_{30}+\beta_{31}\cdot Moderator_{j}+r_{3j}$]. The values by the ovals are standardized regression coefficients of the fixed-effects for each parameter (*β*_00_, *β*_10_, *β*_20_, and *β*_30_), averaged across the separate moderator analyses. The values by the curved arrows are the correlations between fixed-effects in a baseline model (i.e., without moderator variables). Moderators (cross-level interactions; *β*_01_, *β*_11_, *β*_21_, and *β*_31_) included reasoning (verbal, numerical, abstract), personality (five-factor model), mindsets (goal orientations and implicit theories), and emotional intelligence (MSCEIT branches). See Birney et al. (2018) for details of additional covariates that were included.

Using MLM (specifically, linear mixed-effects regression), we modelled four level 1 random-effects, each conditional on the other; as represented in Figure 4, *π*_0_ = the intercept (mean performance), and three slopes, *π*_1_ = attempt number (experience), *π*_2_ = delay-effect (present vs. absent), and *π*_3_ = outflow-effect (constant vs. variable), and considered a range of level 2 moderators of these effects as cross-level interactions. These are schematically represented in Figure 4 (full details of the analyses can be found in Birney et al. 2018).

For current purposes, there are a number of points that would benefit from some explication. First, while we could have used a SEM approach (e.g., Brose et al. 2021), we used a regression model. Attempts, delay, and outflow conditions were regressed on to the penalty score. Thus, the effects estimated for a given variable are conditional on all other variables in the model (as is standard for regression). Second, the fixed effects component of the analysis (i.e., *β* parameters, which, all else equal, are means of respective *π* parameters across individuals) provide weights for a linear composite which best predicts the DV (i.e., the penalty score). However, when these variables are included as random effects, the individuals’ deviations around each fixed effect is explicitly modelled as reflective latent variables, represented as ovals in Figure 4, although in light of our current argumentation, their ontological status as such remains a supposition (Bollen and Diamantopoulos 2017).

Third, in the parlance presented in this paper, *π*_1_, *π*_2_, and *π*_3_ are within-individual contingency variables (of attempts, delay, and outflow, respectively). To explicate, consider the Attempts variable. *β*_10_ represents the mean within-person change in penalty score contingent on number of blocks attempted, averaged across individuals and controlling for level of delay and outflow^7^. A standard interpretation is implied. The standardised regression coefficient, *b*_10_ = −0.31 (as reported in Figure 4) indicates that on average, penalty scores tended to decrease with repeated attempts. Substantively, we interpreted this as a *learning* or *experience* effect. Importantly, in MLM *π*_1*j*_ represents the within-individual *experience* contingency for each of the *J* individuals; and the average of these is the fixed-effect, *β*_10_, as just described. In this study we also considered between-individual differences variables as moderators. Although not represented in Figure 4 for simplicity, in the case of the *experience* contingency, verbal reasoning ability was a statistically significant moderator; the contingency effect of experience was more pronounced for those with higher verbal reasoning scores. Further details of the significant moderators of these parameters are reported in Birney et al. (2018).

If we assume for a moment that we had set this study up to operationalise cognitive flexibility, what aspect of the model would we expect cognitive flexibility to equate to? The traditional approach would suggest that performance after controlling for differences in conditions (e.g., number of attempts, and the delay/outflow effects) would best represent the essence of what is required by the task; this would be the respective mean for each person (*π*_0*j*_). However, the notion of cognitive flexibility that we advocate is not framed in terms of averaging across conditions or holding them constant, rather it is defined in terms of idiosyncratic (within-individual) responses to changing conditions. Thus, a model of intelligence as cognitive flexibility indexed in some way by *π* contingency parameters is needed. There might also be a temptation to define cognitive flexibility as the higher-order reflective factor common to all four latent variables, but this would be short-sighted and premature for the reasons we outline in this paper. 

### 5.3. Summary of Part 4: Why Multilevel Models Are Important

The addition of within-individual process accounts of intelligence as cognitive flexibility introduces the stringent requirement for validity to be established using experimental-psychology methods. First and foremost, we should aim to develop theories for, and seek evidence of a dissociation of level 1 (within-person) process parameters based on theoretically grounded manipulations (e.g., costs and trajectories). Second, evidence of systematic level 2 variability (between-individual) in the theoretically validated level 1 parameters should be obtained. Using this MLM framework, the distinctiveness in processes and the importance of cognitive flexibility is evidenced by four effects. (1) Substantial within-individual variability in trial/item performance; (2) Systematic *within-individual* effects as a function of process manipulations; (3) Substantial between-individual variability in process-effects; and (4) Systematic between-individual effects of *within-individual* effects as a function of *real-world* factors where adaptivity is important. In lieu of *real-world tasks*, appropriately designed dynamic microworlds may be effective (Funke et al. 2018), yet an arbitrary artificialness in even these tasks persists. Evidence in favour of these effects will support our supposition that our understanding of the processes underlying intelligence as *cognitive flexibility* can be enhanced if it is operationalised how it is conceptualised.

## 6. Implications and Final Considerations

During the peer-review process, anonymous reviewers, to whom we express our deep gratitude, raised some interesting discussion points, which we would like to take the opportunity to paraphrase, share, and comment on. As a caveat, and possibly case in point to the challenges our call for reform presents, the attentive reader will notice that in our responses we may have drifted into interpretations and explanations that perpetuate some of the poor practices we have criticised in this paper. For instance, we will discuss CHC factors without questioning their reflective or formative status, and in doing so, we might also be pulled up for assuming ergodicity. For the purpose of communication, we risk this inconsistency.

### 6.1. Beyond Fluid Intelligence: Why Flexibility Is Relevant to Intelligence Generally, and Other CHC Factors

We have framed much of our thinking in terms of fluid intelligence, so a reasonable question is whether our model of within-individual flexibility is limited to *Gf*, and therefore does not apply to intelligence generally? In response, we would argue that broader constructs of intelligence likely have similar within-individual conceptualisations. For instance, if one were to consider intelligence constructs such as Practical Intelligence (Sternberg et al. 2000), Cultural Intelligence (Sternberg et al. 2021), or even Emotional Intelligence (Mayer and Salovey 1993), the notion of within-individual, contingent adaptation is central to their conceptualisation. In fact, the cognitive notion of relational integration extends quite naturally to meaning making from adaptive contingencies (i.e., relational bindings) between goals however defined in a given context and non-cognitive content (emotions, affect), possibly filtered through individual differences in personality dispositions, self-concepts, attitudes and value, and the like (as described in Section 5.1).

In terms of other CHC factors, some are functionally closer to elementary processes that define features of a process account (i.e., as inputs to flexibility). For instance, Jewsbury et al. (2016) demonstrated that processing (mental) speed is largely indistinguishable from the Inhibition process conceptualised in the executive function literature (Miyake and Friedman 2012). Although it might be disputed, the conceptual groupings of broad factors proposed by Schneider et al. (2016, p. 5, Figure 2)—*Perceptual Processing* (e.g., *Ga*, *Gv*, etc.), *Controlled Attention* (e.g., *Gf*, *Gwm*, *Gs*), *Acquired Knowledge* (e.g., *Gc*, *Gq*, *Grw*, *Gr*, *Gl*), and *Psychomotor Abilities* (e.g., *Gp*, *Gps*)—further justify our expectation that the flexibility framework is not relevant to all CHC factors (see Schneider et al. for explanations of abbreviations). For instance, we can set aside Schneider et al.’s Psychomotor Abilities as outside of scope. The Controlled Attention and Perceptual Processing factors are largely process-focused as just described, or *Gf* which we have addressed. This leaves the Acquired Knowledge factors.

Crystalized intelligence (*Gc*) may be seen to presents an interesting challenge to our flexibility account, although if one were to accept the tenets of the *Gf-Gc* Investment Theory, even here the development of *Gc* can be mapped as a series of dynamic, within-person (goal-directed) interactions between the environment and the cognitive and affective resources (processes) one has at their disposal to deal with everyday challenges (Ackerman 1996; Ziegler et al. 2012). The extent to which general encoding (*Gl*) and retrieval (*Gr*) factors draw on historical *Gf* and *Gc*, the same account can be applied. Thus, prima facie, we see no reason to constrain our within-person account to just fluid intelligence at this point, although this is an area ripe for investigations.

### 6.2. Beyond Novelty Processing: Why Flexibility Is Relevant to Routine Reasoning and Cognitive-Capacity

While our case for flexibility is relevant to the novelty aspects common to many definitions of fluid intelligence, it is reasonable to question whether the MLM-contingency framework applies to features/facets of *Gf* that are not inherently to do with novelty, such as routine reasoning in predictable situations, and general cognitive capability. In response, we would argue that in the scheme of one’s overall problem-solving exposure, even routine problems are opportunities to observe flexibility. First, it is interesting to note that we tend not to think of intelligence as a propensity to plod through solving routine, algorithmic problems in routine ways. In such situations we do however give credit for efficiency (e.g., quickly recognising problems are routine), coming up with better (novel) strategies, and doing so with minimal waste of resources. Recognising that a problem is a familiar one (rather than a novel one), drawing on a previously proven solution path (rather than investing effort to create a novel one), and monitoring for possible changes along the way all entails rudimentary adaptation to changing circumstances. Thus, even solution of routine, predictable problems, entails some level of flexibility.

Explanations of reasoning proper (i.e., independent of context) and general cognitive capability (i.e., what it is and how it happens) beyond descriptive accounts remains frustratingly elusive to both experimental and differential psychology. We have already outlined reasons why alone the between-person approach will not help in this regard. Cognitive psychology models, some of which we have reviewed here, go part of the way in presenting a process account of reasoning and general cognitive capability. The lazy (but likely) response is to define reasoning as an emergent property of a system of interacting basic, attentional control, relational binding, and memory processes, with cognitive capability reflected in the efficiency of such a system, often presented as a source of individual differences. Notwithstanding the myriad challenges of this account (or maybe because of them), the need for a within-individual framework of reasoning seems to be amplified rather than diminished. It is our expectation that a formalized, integrated MLM of structurally informed within- and between-person aspects of reasoning, such as the one we have proposed, may provide impetus for a renewed line of investigative efforts.

### 6.3. Beyond Factor-Analysis: Why Methods Matter When Studying Flexibility

While we have critiqued the use of between-person methods, we are not disputing factor analysis as a pragmatic data reduction tool, nor as a measurement tool (especially when framed as a tau equivalent measurement model). We make a distinction between factor analysis as a data-reduction tool, and structural-equation modelling (SEM) generally as a theory testing tool. Multilevel modelling of within- and between-individual differences can be achieved using a range of comparable methods and procedures, including SEM growth-curve models (e.g., ML-SEM, Brose et al. 2021), fixed-link SEM models (Schweizer 2009), or linear mixed-effects regression procedures (Birney et al. 2017; Birney et al. 2018). For those less familiar with the nuance of factor-analytic approaches underlying SEM (e.g., cognitive psychologist interested in individual differences), multilevel regression may be more palatable.

However, we do take issue with the dominant tendency of researchers to use reflective models as the default position without considering alternatives (as evidenced by the status quo, despite compelling arguments of their critical limitation). Furthermore, in our view, the speculation that factor analysis can purify observed test scores from error, and therefore allow one to arrive at an estimate of the supposed-to-be “true” attribute and magnitude of an effect is an unfortunate overuse of factor analysis. The same criticism would apply to an overuse of “shrinkage” in MLM regression models if this was observed to occur. Relying on sophisticated statistical tools to “purify” our measures from what are ultimately method-effects (Birney et al. 2022) reflects how little we understand about the sources of impurity (e.g., unreliability or multidimensionality) in our measures (van der Maas et al. 2017). We would do better to improve our measures using strong theory and better linked conceptual and empirical models, rather than make dubiously justified statistical adjustments. Doing so, we argue, requires building structural hypotheses (Lohman and Ippel 1993) and taking within-person process accounts seriously. In sum, we do not have issues with correlations per se, we have issue with between-person correlations being portrayed (and then interpreted) as the only foundation for the conceptualisation and measurement of intelligence conceived as cognitive flexibility.

Finally, while we are not disputing the pragmatic utility of factor analysis, it is important to understand its mathematical foundations, even if we intend only to use the identified structure merely as a description of the covariation of some set of low-level processes. When we talk about the narrow facets of fluid ability, such as (1) induction, (2) deduction, or (3) sequential reasoning, it is easy to assume that the resulting latent variable reflects an aggregation or accumulation of the separate processes. However, mathematically, this is not the case. When each facet is added to a factor analysis, the derived common factor, which we might label *Gf*, is formally a statistical distillation of what is common in the facets. It is not an aggregation or accumulation of the separate facets. Therefore, when we say fluid intelligence entails, induction, deduction, and sequential reasoning, because they are the indicators (tasks) we have used to “define” the common factor, we have erred. That might be our theoretical explanation, but the common factor is nothing more and nothing less than the very precise thing these three attributes have in common. Our point is, the mathematical derivation of a *Gf* factor is as much an integral part of its operationalisation as are the tasks chosen. If we think otherwise, even as a first step, our methodological basis will be disconnected from the theory to an unknown extent.

### 6.4. Beyond the Status Quo: The Implications of Getting It Wrong

One of the implications of getting it wrong is highlighted by Fried (2020) and also by Protzko (2017). The gist of what is argued by both is that because reflective models assume the components (indicators, markers, manifest variables) are caused by the latent attribute, then the pathway to intervention by targeting indicators is logically precluded. From this sense, the latent variable is an inherent characteristic of the individual. Formative models (specifically causal-formative ones) on the other hand, where the latent variable is caused by the indicators, provide a pathway for intervention. Change the components, and the latent variable will change (that is, in formative models, the latent variable does not exist as an attribute independent of its indicators). For Fried, the impact is on indicators (symptoms) of pathology. If pathology is inherent in the individual, intervening on the symptoms is unlikely to be helpful. For Protzko, the impact is in regard to cognitive training. If intelligence is a reflective latent attribute, with WMC (say) as a reflective indicator (of the impact of intelligence on it), then training WMC is logically precluded from having an impact on intelligence.

It is interesting to note the relatively recent shift from talking about elementary cognitive tasks to elementary cognitive processes. Refined process theories are a good thing for intraindividual accounts. However, we need to be careful that we do not introduce these “processes” simply as a means to take the heat off reflective assumptions made at higher levels. In CHC framing, ECTs serve as indicators of narrow factors. If they are now processes (fundaments) in their own right, evidence for their status as reflective latent variables need to be demonstrated. Additionally, if these fundaments are reflective latent attributes, what is now the status of assumed-to-be reflective latent variables higher in the hierarchy, that is the “broad-factors”. For instance, what is the status of *Gf* (an abstraction of narrow ability factors) in models already made up of reflective latent processes defined at the lowest level, keeping in mind the variance distillation that occurs in factor analysis we have just pointed out. The notion of *Gf* as a causal-formative umbrella of lower-order reflective attributes becomes not only plausible, but possibly, logically necessary. For many, framing *Gf* as a formative variable is a step too far. It seems an elegant resolution may be to move beyond the simple common-process account of factor analysis, and instead invest resources into further investigations and development of time-varying network models and directed acyclic graphs (Fried 2020).

### 6.5. Conclusion

We accept as historical fact the dominant, foundational psychometric approach to intellectual abilities as that which started more or less around the time of Charles Spearman (circa 1900) and led to the Cattell-Horn-Carroll (CHC) hierarchical taxonomy. As noted by Conway et al. (2021, p. 6), CHC is a “model of the covariance structure of cognitive abilities… but it is not a psychological theory”. Historically, establishing the validity of constructs like intelligence has been dominated by considerations of a nomological network of convergent and divergent correlations. In this conceptual analysis and review, we first considered implications of the supposition of stability as antithetical to variability, along with the ergodic claim that between-person models can be extended to within-person processes. We also considered the dominance of reflective common-factor conceptualisations and the neglect and subsequent dismissal of formative ones (Kovacs and Conway 2016; van der Maas et al. 2006; van der Maas et al. 2017). We explicated causal-formative accounts, in contrast to composite-formative ones (Bollen and Diamantopoulos 2017), as relevant to our goal to explicate a within-individual perspective of intelligence. This is because causal accounts put process and mechanism within the realm of direct observation through experimental manipulation and explicit process accounts, rather than leaving them to be inferred and reified from patterns of discriminant and convergent correlations after data have been collected. However, regardless of whether one studies formative or reflective concepts, or even network models, the burden of process identification is the responsibility of the researcher and cannot be delegated to statistics, no matter their levels of sophistication (Birney et al. 2022).

The promise of working memory theory to provide an explanatory account of intelligence (or at least intelligence test performance) has not been missed by intelligence researchers (Carpenter et al. 1990; Daneman and Carpenter 1980). There has been considerable debate regarding the dissociation of WM and intelligence (Ackerman et al. 2005; Blair 2006; Guthke et al. 2003; Kyllonen and Christal 1990). While more and more refined accounts of WM processes have been developed (Oberauer 2021), some we have reviewed here, these have not been matched by similarly well-honed accounts of intelligence. If anything, the limitations of traditional ways to conceptualise processes underlying intelligence and the inertial resistance to new approaches have only been amplified over time. We are of the view that much can be achieved by advancing the alignment of conceptual definitions and methodological considerations which build on modelling within-person variability.

In sum, we have four recommendations, (1) do not assume stationarity, test for it, (2) recognize within-individual (process) accounts are critical to understanding individual differences, (3) be wary of using reflective models as a starting point for theory development, and (4) multilevel models are a good for theory development, and for specifying and testing structural hypotheses regarding within-individual and between-individual differences and their moderators.

## Figures and Tables

**Table 1:** Examples contingent (Level 1) and moderating (Level 2) indicators of cognitive flexibility.

Level 1 *X* Factors Vary across Occasion and Individuals	Level 1 *Z* Factors Vary across Occasion, Constant across Individuals	Level 2 Moderators Invariant across Occasion, Vary across Individual
Metacognitive processes Confidence in item response State personality Perception of task/situation demands Perception of feedback etc.	Time (chronological) item-sequence (as an experiential factor) Item complexity (RI demand) Presence of feedback Situation^5^ etc.	Personality traits Working-memory Relational integration ability Age Knowledge/experience etc.

## Data Availability

Not applicable.

## Notes

1. The (typically) unquestioned use of the term “manifest variables” to label observed variables is testament to the assumption that individual differences in scores on these variables are the outward *manifestation* of concomitant individual differences in the latent attribute.

2. While we prefer to reserve the term “measure” for variables where fundamental measurement properties have been demonstrated (see Michell 1990), in our view, conceiving them as latent variables that happen to have a useful coding metric is more appropriate (see Birney et al. 2022).

3. The trait-complex example also serves to demonstrate a second point made by Bollen and Diamantopoulos (2017), that reflective latent variables (e.g., extraversion, when appropriately conceived of) can act as composite-formative indicators in other models (such as of trait-complexes).

4. This is also not to say that with greater understanding, the status of concepts will necessarily move from formative to reflective. Some concepts, maybe most, are by nature and definition, formative.

5. According to Beckmann (2010; see also Birney et al. 2016) within the framework of person-task-situation interactions, the situation refers to the context or circumstances in which a task is performed. It constitutes a source of complexity in addition to the processing demands posed by the task itself and therefore contributes to the overall complexity and consequently impacts performance. The user-interface, the clarity of instructions, time pressure, or the semanticity of variable labels in a CPS system are examples for such situation components.

6. Of course, multidimensionality introduces other challenges to measurement that would need to be explicated in the theoretical model.

7. As well as covariates, as mentioned previously.
